# Mapping of the minimal inorganic phosphate transporting unit of human PiT2 suggests a structure universal to PiT-related proteins from all kingdoms of life

**DOI:** 10.1186/1471-2091-12-21

**Published:** 2011-05-17

**Authors:** Pernille Bøttger, Lene Pedersen

**Affiliations:** 1 Department of Molecular Biology, Aarhus University, C. F. Møllers Allé 3, Aarhus C, DK-8000, Denmark; 2 Institute of Clinical Medicine, Aarhus University, Brendstrupgårdsvej 100, Aarhus N, DK-8200, Denmark; 3 Department of Haematology, Aarhus University Hospital, Tage-Hansens gade 2, DK-8000 Aarhus C, Denmark; 4 Department of Medical Biochemistry, Ole Worms Allé 3, Aarhus University, DK-8000 Aarhus C, Denmark

## Abstract

**Background:**

The inorganic (P_i_) phosphate transporter (PiT) family comprises known and putative Na^+^- or H^+^-dependent P_i_-transporting proteins with representatives from all kingdoms. The mammalian members are placed in the outer cell membranes and suggested to supply cells with P_i _to maintain house-keeping functions. Alignment of protein sequences representing PiT family members from all kingdoms reveals the presence of conserved amino acids and that bacterial phosphate permeases and putative phosphate permeases from archaea lack substantial parts of the protein sequence when compared to the mammalian PiT family members. Besides being Na^+^-dependent P_i _(NaP_i_) transporters, the mammalian PiT paralogs, PiT1 and PiT2, also are receptors for gamma-retroviruses. We have here exploited the dual-function of PiT1 and PiT2 to study the structure-function relationship of PiT proteins.

**Results:**

We show that the human PiT2 histidine, H_502_, and the human PiT1 glutamate, E_70_, - both conserved in eukaryotic PiT family members - are critical for P_i _transport function. Noticeably, human PiT2 H_502 _is located in the C-terminal PiT family signature sequence, and human PiT1 E_70 _is located in ProDom domains characteristic for all PiT family members.

A human PiT2 truncation mutant, which consists of the predicted 10 transmembrane (TM) domain backbone without a large intracellular domain (human PiT2ΔR_254_-V_483_), was found to be a fully functional P_i _transporter. Further truncation of the human PiT2 protein by additional removal of two predicted TM domains together with the large intracellular domain created a mutant that resembles a bacterial phosphate permease and an archaeal putative phosphate permease. This human PiT2 truncation mutant (human PiT2ΔL_183_-V_483_) did also support P_i _transport albeit at very low levels.

**Conclusions:**

The results suggest that the overall structure of the P_i_-transporting unit of the PiT family proteins has remained unchanged during evolution. Moreover, in combination, our studies of the gene structure of the human PiT1 and PiT2 genes (*SLC20A1 *and *SLC20A2*, respectively) and alignment of protein sequences of PiT family members from all kingdoms, along with the studies of the dual functions of the human PiT paralogs show that these proteins are excellent as models for studying the evolution of a protein's structure-function relationship.

## Background

Phosphate is needed by any living cell for structural and metabolic purposes. Inorganic phosphate (P_i_) has to be actively transported across the cell membrane against a chemical and electrical gradient. In mammalian cells this task is managed by the type III sodium-dependent P_i _(NaP_i_) symporters, PiT1 and PiT2, which utilize the free energy provided by the Na^+ ^concentration gradient as the driving force for uphill import of P_i_[1-3], reviewed in [4].

The mammalian type III transporters are part of the P_i _transport (PiT) family (SLC20 [5]; TC #2.A.20 [6]), but several members were originally identified as receptors for different retroviruses belonging to the gamma-retrovirus genus [7-13]; thus, PiT1 and PiT2 are proteins with dual functions. The PiT family also comprises non-mammalian members, e.g., fungus Pho-4^+ ^(*Neurospora crassa *(*N. crassa*)) [14] and yeast Pho89 (*Saccharomyces cerevisiae *(*S. cerevisiae*)) [15] as well as the proton (H^+^)-dependent P_i _transporters from bacteria, PiTA and PiTB (*Escherichia coli *(*E. coli*)) [16], and plant Pht2_1 (*Arabidopsis thaliana *(*A. thaliana*)) [17]. Furthermore, there is an increasing number of entries in the National Center for Biotechnology Information (NCBI) protein database (URL: http://www.ncbi.nlm.nih.gov/) that show similarity to the known members of the PiT family and therefore are denoted putative phosphate permeases; and PiT family members have been found in all kingdoms [18], reviewed in [19]. Altogether, this suggests that the PiT proteins developed very early in evolution and that this family of proteins has important function(s) in all kingdoms of life.

**Figure 1:**
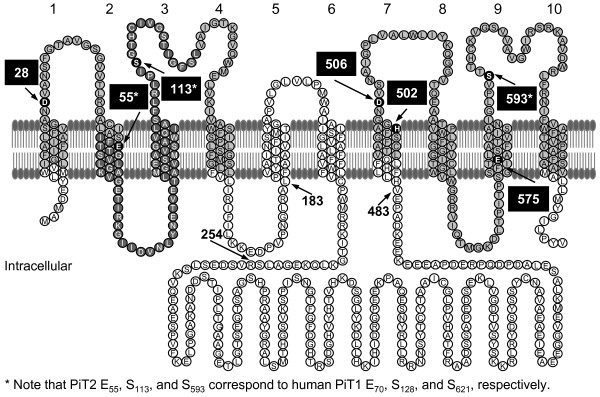
**Putative topological model of human PiT2 and mutants**. Putative membrane topology model of human PiT2 on which the mutant proteins investigated in the present paper are based; the model was originally proposed by O'Hara and coworkers [8,20]. The numbers of the TMs are indicated above the model. Other membrane topology models have been proposed for PiT1 [22,23] and PiT2 [24], which suggested diverging topology for the two paralogs; the alternative PiT2 model is shown in Figure 2. The amino acids previously identified in human PiT2 as being critical for P_i _transport function are highlighted with black filling and pointed out with arrows; D_28_, E_55_, S_113_, D_506_, E_575_, and S_593_[18,27,28]. In human PiT1, the amino acids S_128 _(PiT2 S_113_) and S_621 _(PiT2 S_593_) have previously been identified as being critical for PiT1 P_i _transport function [29]. In the present study, human PiT2 H_502 _(situated in the PiT family signature sequence) and human PiT1 E_70 _(equivalent in position to human PiT2 E_55_) are also identified as critical for P_i _transport function (see Figure 3). The grey-filled sequences (L_11_-L_161 _and V_492_-V_640_), represent the N- and C-terminal, respectively, ProDom domains (PD001131) published in 2004 defining the PiT family members [27]. The dark grey-filed sequence (I_53_-L_127_) represents the most recent ProDom domain defining the PiT family members http://prodom.prabi.fr/.

**Figure 2:**
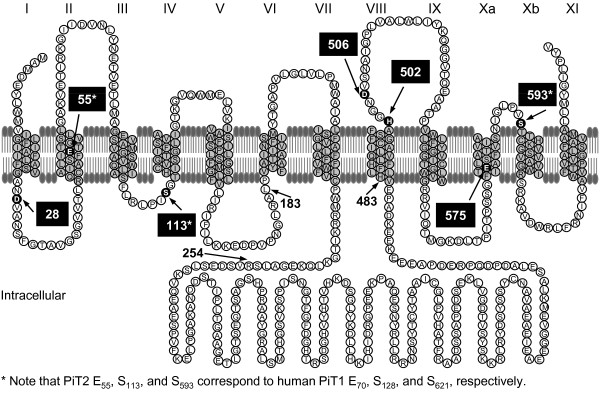
**Alternative topological model of human PiT2 and mutants**. Membrane topology model for human PiT2 suggested by Salaün and coworkers [24]; the TMs are shown as grey-filled sequences and their numbers are indicated with roman numbers above the model. This model shares some similarity to a membrane topology model for PiT1 proposed in 2002 [22]. Based on the cellular location of C-terminal tags, the C-terminal ends of PiT1 and PiT2 were predicted to be extracellular [22,24]. And based on the cellular location of an N-terminal tag on PiT2 and glycosylation of a site in human PiT1 and partly glycosylation of the same site in human PiT2 although oddly not in hamster PiT2, the N-termini of PiT1 and PiT2 were suggested to be extracellular [22,24]; due to a suggested additional TM after TM3 in Figure 1 (TMIV in this figure), this did not influence the orientation of the large intracellular domain in these models compared to the model in Figure 1. The PiT2 model shown in Figure 1 and this figure, respectively, and the PiT1 model proposed in 2002 [22] were later compared by us [18]. In 2009, Farrell and coworkers proposed a modified model of human PiT1 based on substituted cysteine accessibility mutagenesis [23]. The recent model of PiT1 shows more resemblance to the PiT2 models shown in this figure and in Figure 1 concerning the length and position of the large intracellular domain (L6) than the model from 2002. The amino acids identified in human PiT2 and human PiT1 as being critical for P_i _transport function are highlighted with black filling and pointed out with arrows; for references see legend to Figure 1. Compared to the PiT2 model in Figure 1, the PiT2 model proposed by Salaün and coworkers (this figure) and the PiT1 model proposed in 2009 by Farrell and coworkers do not affect the placement of PiT1 D_43 _(PiT2 D_28_), PiT1 E_70 _(PiT2 E_55_), PiT1 H_530 _(PiT2 H_502_), PiT1 D_534 _(PiT2 D_506_), PiT1 E_603 _(PiT2 E_575_), and PiT1 S_621 _(PiT2 S_593_) in either a TM domain or a loop sequence [23,24] (compare Figure 1 and this figure). However, PiT1 S_128 _(PiT2 S_113_) placed in loop regions in the PiT2 models (this figure and Figure 1), is in the PiT1 model from 2009 suggested to be placed in a TM domain [23].

The first membrane topology model of PiT proteins was based on Kyte-Doolittle hydropathy plots. Analyses of human PiT1 and Pho-4^+ ^protein sequences predicted 10 transmembrane (TM) domains, 9 loops (here referred to as L1 to L9) hereof 5 extracellular, internal N- and C-terminal ends, and a large hydrophilic domain (L6) intracellularly positioned between the putative 6^th ^and 7^th ^TM domains [20]. Due to profound similarity (approx. 62% amino acid identity) between the human PiT paralogs, the same membrane topology model was proposed to also apply for human PiT2 (Figure 1) [8]. The model was, moreover, supported by the experimental assignment of the large intracellular domain of rat PiT2 to the cytoplasmic space [21]. Other topology models have, however, been proposed for PiT1 [22,23] and PiT2 [24] (Figure 2); please see legend to Figure 2 for more details. Nevertheless, we have shown that exchanging as little as 12 or 15 amino acids in the fungal PiT protein, Pho-4^+^, with human PiT1 or human PiT2 sequences, respectively, results in proteins that support infection by human PiT1 or PiT2 cognate gamma-retroviruses [25,26]; results, which suggest that these transporters are structurally highly related.

Analyzing human PiT1 and Pho-4^+ ^sequences, Johann and coworkers discovered an internal sequence repeat, which they suggested had originated from an ancient gene duplication [20]. Both regions were shown to harbor a ProDom domain, PD001131 (Figure 1) [27] (human PiT2 I_11_-L_161 _and V_492_-V_640_), characteristic for all members of the PiT family. Interestingly, all amino acids in human PiT2 (i.e. D_28_, E_55_, S_113_, D_506_, E_575_, and S_593_) and in human PiT1 (S_128 _and S_621_) identified to be critical for P_i _transport function are located in these ProDom domains (Figure 1) [18,27-29]. It should be noted, that, the ProDom domain PD001131 has changed and now consists of what corresponds to human PiT2 I_53_-L_127 _http://prodom.prabi.fr (Figure 1). In an attempt to narrow down a PiT family trait, Saier aligned the N-terminal protein sequences from 17 members representing all kingdoms [6,30]. The author noted the existence of an 11-amino-acid-long sequence in the N-terminal region containing the conserved core sequence [GANDVANA] and proposed it to be a signature sequence for the PiT family [6,30]. However, refined studies of the N- and C-termini of 109 protein sequences representing PiT family members from all kingdoms revealed that these proteins harbor a 12-amino-acid-long PiT family signature sequence - with the common core consensus sequence [GANDVANA] - within each of the PD001131 ProDom domains proposed in 2004 [18]. Furthermore, D_28 _and D_506 _shown to be critical for PiT2 P_i _transport are placed in either of the PiT family signature sequences [18].

To further investigate the importance of the PiT family signature sequences, we have analyzed the human PiT2 histidine, H_502_, located in the C-terminal PiT family signature sequence, and we show that it is indeed critical for the P_i _transport function but dispensable for infection by PiT2 cognate gamma-retroviruses. The human PiT2 H_502 _is the second amino acid in this sequence to be identified as critical for P_i _transport function. In addition, we also show that the human PiT1 glutamate, E_70_, located in the PD001131 ProDom domain, is critical for the P_i _transport function but dispensable for infection by PiT1 cognate gamma-retroviruses.

We have, moreover, combined studies of the gene structure of the human PiT genes (*SLC20A1 *and *SLC20A2*), alignment and TM domain prediction of protein sequences of PiT family members from all kingdoms of life, and studies of the dual functions of the human PiT paralogs as P_i _transporters and gamma-retroviral receptors, and we found that these proteins are excellent as models for studying the evolution of protein structure-function relationship. Specifically based on the observation that bacterial and archaeal PiT family members are substantially smaller than eukaryotic members [18] and our alignment (Additional File 1 Figure A), we analyzed truncation mutants of human PiT2. Our results clearly show that the large intracellular domain of human PiT2 is dispensable for P_i _transport function, and that a fully functional P_i_-transporting unit can be created by the 10 TM domains and the small loop sequences connecting them (human PiT2ΔR_254_-V_483_). A further truncated human PiT2 protein with the 5^th ^and 6^th ^TM domains and the large intracellular domain removed resembles the structures of as well a putative phosphate permease from archaea as of PiTA from bacteria (*Archaeoglobus fulgidus *(*A. fulgidus*) and *E*. *coli*, respectively). This mutant (human PiT2ΔL_183_-V_483_) was an excellent gamma-retroviral receptor [31], and we here show that it can support low levels of P_i _transport. Altogether, these results suggest that the overall structure of the P_i_-transporting unit of the PiT family proteins has remained unchanged during evolution.

## Methods

### Sequence alignment

Protein sequence alignment of nine PiT family members representing all kingdoms was made using the ClustalW alignment program version 2.0.12 available at the European Bioinformatics Institute server (URL: http://www.ebi.ac.uk/clustalw2/) [32]. The Swiss-Prot protein sequences were retrieved from the NCBI Protein server (URL: http://www.ncbi.nlm.nih.gov/protein/). Accession numbers are: *Homo sapiens *(*H. sapiens*) PiT2 [Swiss-Prot:Q08357], *H. sapiens *PiT1 [Swiss-Prot:Q08344], *Caenhorabditis elegans *(*C. elegans*) putative phosphate permease [Swiss-Prot:Q17455], *Drosophila melanogaster *(*D. melanogaster*) putative phosphate permease [Swiss-Prot:Q9VTG0], *N. crassa *Pho-4^+ ^[Swiss-Prot:P15710], *Trypanosoma brucei *(*T. brucei*) putative phosphate permease [Swiss-Prot:Q9N930], *A. fulgidus *putative phosphate permease [Swiss-Prot:O29467], *A. thaliana *Pht2_1 [Swiss-Prot:Q38954], and *E. coli *PiTA [Swiss-Prot:P37308]. All sequences encompass two 12-amino-acid-long sequences, which based on comparison of 109 sequences, were identified in PiT proteins and related proteins and suggested to be PiT family signature sequences [18]. We, however, observed that the C-terminal PiT family signature sequence of *E. coli *PiTA did not group together with the C-terminal PiT family signature sequences of the eight other species in the alignment (data not shown). In order to group all the C-terminal PiT family signature sequences together, the alignment was adjusted manually after an alignment of *H. sapiens *PiT2 amino acids S_422_-V_652 _[Swiss-Prot:Q08357], *A. fulgidus *putative phosphate permease [Swiss-Prot:O29467], and *E. coli *PiTA [Swiss-Prot:P37308]. For the adjusted protein sequence alignment of the PiT family members, see Additional File 1 Figure A.

### Prediction of TM domains in the PiT family members and related proteins

Putative TM domains were predicted using the TMHMM Server v. 2.0 available at the Center for Biological Sequence Analysis, Technical University of Denmark (URL: http://www.cbs.dtu.dk/services/TMHMM/), and the Dense Alignment Surface (DAS) Transmembrane Prediction server available at the Stockholm Bioinformatics Center, Stockholm University (URL: http://www.sbc.su.se/~miklos/DAS/). TMHMM is based on a hidden Markov model (HMM) that is cyclic with seven types of states for helix core, helix caps on either side, loop on the cytoplasmic side, two loops for the non-cytoplasmic side, and a globular domain state in the middle of each loop [33], and DAS is based on low-stringency dot-plots of the query sequence against a collection of non-homologous membrane proteins using a previously derived special scoring matrix [34].

In general, the predictions using both servers correspond well to each other when compared (data not shown), however, the DAS server tends to predict shorter TM domains in agreement with the tendency for prokaryotic TM domains to be shorter in length when compared to the length of eukaryotic TM domains [35]. Therefore, we chose to use the DAS server over the TMHMM server when predicting TM domains in the prokaryotic protein sequences for *E. coli *PiTA and *A. fulgidus *putative phosphate permease. The predicted TM domains are shown in Additional File 1 Figure A.

### Intron-exon border analysis of human PiT genes *SLC20A1 *and *SLC20A2*

The SPIDEY mRNA-to-genome DNA alignment program version 1.40 available from the NCBI homepage (URL: http://www.ncbi.nlm.nih.gov/spidey/index.html) [36] was used to determine the location of intron-exon borders in the human PiT genes. SPIDEY takes as input an mRNA sequence and the corresponding genomic sequence, and it generates an alignment that establishes the gene structure. The GenBank mRNA sequences were retrieved from the NCBI nucleotide server (http://www.ncbi.nlm.nih.gov/nuccore/). Accession numbers are: *H. sapiens *PiT1 mRNA [GenBank:NM_005415] and *H. sapiens *PiT2 mRNA [GenBank:NM_006749]. The genomic GenBank sequences were retrieved from the NCBI human genome server (http://www.ncbi.nlm.nih.gov/projects/genome/guide/human/). Accession numbers are: *H. sapiens *chromosome 2 (*SLC20A1*) [GenBank:NC_000002] and *H. sapiens *chromosome 8 (*SLC20A2*) [GenBank:NC_000008]. The intron-exon borders are shown in Additional File 1 Figure A on the protein sequence alignment of nine PiT family members.

### Expression plasmids

The pcDNA1A^R^tkpA-derived expression plasmids pOJ74 and pOJ75 (Wyeth-Ayerst Research, Pearl River N.Y., USA) encoding human PiT2 and PiT1, respectively, have been described [37].

The plasmid encoding the human PiT2 H_502_A mutant was made by using the QuickChange^® ^XL site-directed mutagenesis kit (Stratagene, La Jolla CA, USA) according to the manufacturer's instructions. Besides the mutations creating H_502_A, the forward primer 5'-TTCGGGTCCTTTGCTGCCGGCGGCAATGACGT-3' and reverse primer 5'-ACGTCATTGCCGCCGGCAGCAAAGGACCCGAA-3' also generated, by introduction of a silent mutation, an *NgoM *IV restriction enzyme cleavage site in pOJ74, which was used for screening. The plasmid encoding the human PiT1 E_70_K mutant was made by using the Altered sites II kit (Promega, Madison WI, USA) according to the manufacturer's instructions. A mutation creating E_70_K as well as a *Dra *I restriction enzyme cleavage site was introduced into a pAlter-1 vector (Promega) harboring the *Pst *1 - *Hind *III fragment of pOJ75 (the nucleotide sequence encoding the N-terminal part of the human PiT1 protein) using the primer 5'-GACAGAGCCCACTGTTTTAAAGATGCTAGCTAG-3'. Finally, this construct was digested with *Kpn *I and *Hind *III generating a fragment, which was used to replace the corresponding fragment in pOJ75 resulting in the desired plasmid.

The plasmid encoding the human PiT2ΔL_183_-V_483 _mutant has previously been described [31]. The plasmid encoding the human PiT2ΔR_254_-V_483 _mutant was made using a pAlter-1 vector harboring the *Pst *I - *Hind *III fragment of pOJ74 (the nucleotide sequence encoding the N-terminal part of the human PiT2 protein) as template in a polymerase chain reaction (PCR) with the forward primer 5'CTATAGGGAGACCCAAGCTTTGTTTATTTAA3' and the reverse primer 5'GAGGACCTGGAGGAAATGGAACAGGAGGTGTGATAAAGCACCTTCTTTTTG3'; the latter primer was used to create the link between the 5' sequence encoding KEGALS_253 _and the 3' sequence encoding H_484_LLFH (Figure 1). The amplification product was digested with *Sse *8387 I and *Hind *III and used to replace the corresponding fragment in pOJ74 resulting in the desired plasmid.

The authenticities of all the nucleotide sequences were confirmed.

The plasmids were purified using either cesium chloride (CsCl) according to the protocol described by Maniatis and coworkers [38], or using Nucleobond (Macherey-Nagel, Düren, Germany) or Qiagen maxiprep (Qiagen GmbH, Hilden, Germany) according to the manufacturer's instructions.

### Cell cultures

Chinese hamster ovary K1 cells, CHO K1 (ATCC CCL-61) and dog osteosarcoma cells, D17 (ATCC CCL-183), were cultivated as described [37]; *Mus dunni *tail fibroblasts, MDTF (ATCC CCL-2017) were cultivated in Dulbecco's modified Eagle's medium supplemented with 10% fetal bovine serum (FBS), 100IU per mL of penicillin (P), and 100 μg of streptomycin (S) per mL (D-MEM/FBS/PS). A-MLV (4070A isolate), 10A1 MLV, and Gibbon ape leukemia virus (GALV, SEATO) pseudotypes of the β-galactosidase-encoding transfer vector G1BgSvN [39] were obtained from the producer cell lines PA317GBN, PT67GBN, and PG13GBN, respectively [40-42]. PT67GBN was established as described [28]. All packaging cells were cultivated in DMEM supplemented with 10% newborn calf serum (NCS) and PS (D-MEM/NCS/PS). Feline leukemia virus subgroup B (FeLV-B) vector pseudotypes carrying the G1BgSvN transfer vector were made essentially as described [43]. Vectors were harvested as supernatants from confluent producer cells, and the vector containing supernatants were filtered (0.45-μm pore size) and stored at -80°C.

### Transient transfection and infection assay

Transient transfection-infection assays were performed essentially as described [37]. Briefly, CHO K1 cells seeded in 60-mm-diameter dishes at 8 × 10^4 ^cells per dish were transfected with 2 μg per dish of plasmid DNA encoding human PiT2 (pOJ74), human PiT1 (pOJ75), human PiT2 H_502_A, human PiT1 E_70_K, or equimolar amounts to human PiT2 of human PiT2ΔR_254_-V_483 _or human PiT2ΔL_183_-V_483_. Mock treated cells were transfected with empty vector DNA (pcDNA1A^R^tkpA). Three independent precipitates were made per construct. Forty-eight hours after transfection, approx. 4 to 8 × 10^4 ^10A1 MLV or A-MLV pseudotypes carrying the G1BgSvN transfer vector were added per dish in the presence of Polybrene. Forty-eight hours later, the dishes were stained and evaluated. Infection was analyzed by counting the number of β-galactosidase-positive (infected) cells per dish. Analyses for FeLV-B and GALV receptor functions were performed on MDTF cells using 1.5 × 10^4 ^cells and approx. 1.5 to 3.0 × 10^4 ^vector pseudotypes per dish. Numbers of vector pseudotypes used in the experiments were calculated from the number of β-galactosidase-positive colonies per mL obtained on D17 cells as described [37].

### ^32^P_i _transport assay

**Figure 3:**
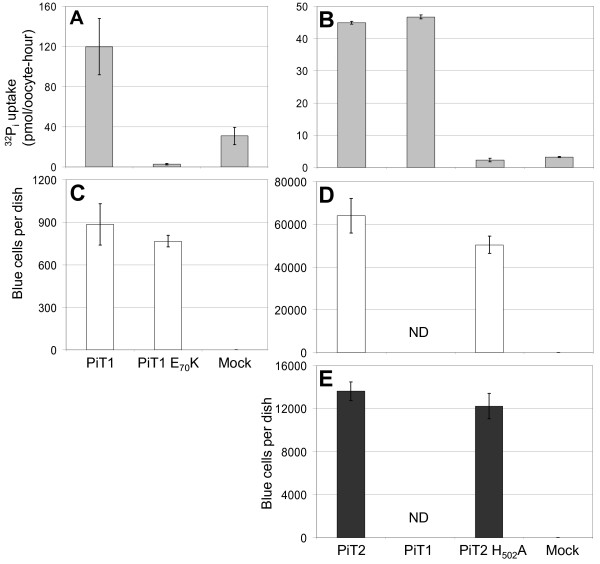
**Analysis of human PiT1 E_70_K and PiT2 H_502_A for Na^32^P_i _uptake and gamma-retroviral receptor function**. A-B *X. laevis *oocytes were injected with H_2_O (Mock) or cRNA of the indicated constructs. Three days later, a ^32^P_i _uptake assay was performed and the ^32^P_i _uptake in individual oocytes was measured. Data are the mean value of (n) numbers of oocytes ±SEM, see Additional File 2 for data and statistics. Experiments A and B were made independently of each other, and the experiments were repeated and similar results obtained. C CHO K1 cells were transfected with CsCl-purified PiT1- or PiT1 E_70_K-encoding plasmid or empty vector DNA (Mock). Three independent precipitates were made for each construct. Forty-eight hours after transfection, approx. 8 × 10^4 ^10A1 MLV pseudotypes were added per dish. The average numbers (±SEM) of blue (infected) cells per dish from three dishes receiving independent precipitates are shown, see Additional File 2 for data and statistics. D-E were made in parallel using the same protocol as in (C) with the exception that Nucleobond-purified plasmids encoding PiT2, PiT2 H_502_A, or empty vector DNA were used. The dishes were challenged with approx. 4 × 10^4 ^10A1 MLV pseudotypes (D) or A-MLV pseudotypes (E). The average numbers (±SEM) of blue (infected) cells per dish from three dishes receiving independent precipitates are shown, see Additional File 2 for data and statistics.

Female *Xenopus laevis (X. laevis) *frogs were obtained from Nasco (Nasco, Modesto CA, USA) and kept and handled according to guidelines from the Danish Animal Experiments Inspectorate. Oocytes were isolated from frogs anesthetized in a 0.1-0.2% MS.222 (3-aminobenzoic acid ethyl ester) (Sigma, St. Louis MO, USA) solution for 10-30 minutes. A 1-1.5 centimeters incision was made in the abdomen and several ovaries were removed surgically by authorized personnel. The oocytes were manually dissected and subsequently collagenase (Sigma, St. Louis MO, USA) treated and maintained in modified Barth's solution [88 mM NaCl, 1 mM KCl, 0.82 mM MgSO_4_, 0.4 mM CaCl_2_, 0.33 mM Ca(NO_3_)_2_, 2.4 mM NaHCO_3_, 10 mM HEPES-KOH, pH 7.5, 100 IU per mL penicillin, 100 μg per mL streptomycin] at 18°C as described [28]. The following day, the oocytes were used for cRNA injection and subsequent analyses of ^32^P_i _uptake essentially as described previously [28]. Briefly, cRNAs were prepared from *Apa *1 (Figure 3A) or *Bln *1 (Figures 3B and 6) linearized plasmid preparations applying the mMESSAGE mMACHINE kit (Ambion, Austin TX, USA). Stage V-VI oocytes were microinjected with 12.5 ng of cRNA (or H_2_O as negative control) and incubated at 18°C. After two to three days, the oocytes were washed in phosphate-free uptake solution [100 mM NaCl, 2 mM KCl, 1 mM CaCl_2_, 1 mM MgCl_2_, 10 mM HEPES-Tris pH 7.5], and hereafter incubated in uptake solution containing 0.1 mM KH_2_^32^PO_4 _(2 mCi per mL, New England Nuclear, Boston MA, USA) at RT for 1 hour. The oocytes were washed in ice-cold uptake solution containing 5 mM KH_2_PO_4 _and the ^32^P_i _uptake of each oocyte measured in a liquid scintillation counter as described previously [28]. It should be noted that factors coupled to the health and husbandry of the female *X. laevis *frogs can influence the oocyte batches. These factors include nutrition, season of the year (light cycle), water temperature, salinity and hardness of the water, water contaminants or toxins, and diseases [44], and the impact is that different batches of oocytes injected with cRNAs encoding the same proteins exhibit different average transport capacities.

### Statistical analysis

The null hypothesis that two mean values are identical was tested by a two-tailed Student's *t*-test. The test compares the actual difference between two mean values in relation to the variation in the data (expressed as the standard error of the difference between the mean values). The null hypothesis was rejected, e.g., the mean values were considered different when *P*<0.05.

## Results and discussion

### Human PiT1 E_70 _and human PiT2 H_502 _are critical for P_i _transport function but dispensable for gamma-retroviral receptor function

In a former study, we identified the putative 2^nd^-TM domain-positioned human PiT2 E_55 _as being critical for PiT2 P_i _transport function (Figure 1) [28]. The human PiT2 paralog, human PiT1, harbors a corresponding glutamate in position 70, E_70_. To investigate whether this conserved residue was important for PiT1 P_i _transport function, it was mutated to a lysine generating the mutant human PiT1 E_70_K. In the experiment shown in Figure 3A, oocytes injected with cRNA encoding human PiT1 supported a ^32^P_i _uptake of 119.86 ±28.16 pmol/oocyte-hour at pH 7.5 in agreement with previous results obtained addressing the Na^32^P_i _uptake function of human PiT1 in *X. laevis *oocytes [45]. The P_i _transport function of human PiT1 E_70_K was severely impaired when compared to that of wildtype PiT1 (*P *= 0.002, 2.78 ±0.74 pmol/oocyte-hour (PiT1 E_70_K)) (Figure 3A); see Additional File 2 for data and statistics to Figure 3.

Besides being P_i_-transporting proteins, the mammalian PiT proteins also serve as gamma-retroviral receptors, and this dual-function allows for analyzing whether a mutated PiT protein is properly processed, folded and translocated to the cell surface [18,28]. The human PiT1 E_70_K mutant was therefore analyzed for gamma-retroviral receptor function using a transient transfection-infection assay [37]. For the infection assay, retroviral vectors harboring a β-galactosidase encoding transfer vector and carrying viral surface proteins responsible for receptor recognition were used; vectors carrying, e.g., 10A1 MLV surface proteins are referred to as 10A1 MLV vector pseudotypes. Eukaryotic expression plasmids encoding human PiT1 and human PiT1 E_70_K mutant protein were transfected into CHO K1 cells non-permissive for infection by 10A1 MLV vector pseudotypes (Figure 3C) [28]. The abilities of these proteins to support infection by 10A1 MLV vector pseudotypes were analyzed; the infection levels were evaluated as the number of β-galactosidase positive (blue) cells per 60-mm-diameter dish. CHO K1 cells expressing human PiT1 were permissive for infection by 10A1 MLV vector pseudotypes (Figure 3C) in agreement with PiT1's well-described receptor function for 10A1 MLV [10]. Moreover, the human PiT1 E_70_K mutant supported wildtype PiT1 levels of 10A1 MLV infection (884 ±146 blue cells per dish (PiT1), 767 ±42 blue cells per dish (PiT1 E_70_K), *P *= 0.48) (Figure 3C). Besides being a receptor for 10A1 MLV, PiT1 is also a receptor for GALV [7] and for FeLV-B [13]. The human PiT1 E_70_K protein was analyzed in parallel for receptor function for vector psedotypes of these two viruses in non-permissive *Mus dunni *tail fibroblasts and found to sustain wildtype PiT1 infection levels of GALV (2087 ±780 blue cells per dish (PiT1), 1992 ±273 blue cells per dish (PiT1 E_70_K), *P *= 0.91) and FeLV-B (1424 ±346 blue cells per dish (PiT1), 1715 ±527 blue cells per dish (PiT1 E_70_K), *P *= 0.67). The wildtype receptor functions of PiT1 E_70_K confirm that the overall membrane topology is preserved and that the processing to the cell surface was unaffected by the E_70_K-mutation.

The glutamate E_70 _in human PiT1 is conserved in eukaryotic PiT family members as are the other two human PiT1 residues (S_128 _and S_621_) (Additional File 1 Figure A) previously shown to be critical for P_i _transport function [29]. Since the corresponding glutamate and serine residues in human PiT2 have already been identified as being critical for P_i _transport function [27,28], this demonstrate that equivalent glutamate or serine residues in the human PiT paralogs both are critical for their P_i _transport functions. These observations illustrate that it is highly likely that the other conserved amino acids identified in human PiT2 as being critical for P_i _transport function also are important for the transport function of human PiT1 and other PiT family members.

The histidine residue, human PiT2 H_502 _is positioned in the 7^th ^TM domain according to the Johann topology model (Figure 1) [20]. It is, moreover, located in the C-terminal PiT family signature sequence and conserved in eukaryotic PiT family members [18] (Additional File 1 Figure A). Moreover, analysis of 60 sequences of bacterial PiT family members revealed only 5 sequences without the histidine residue illustrating that this residue is also highly preserved in the C-terminal PiT family signature sequence of PiT family members belonging to this kingdom [18] (Additional File 1 Figure A). Since the conserved aspartic acid in the C-terminal PiT family signature sequence, that is human PiT2 D_506_, is critical for P_i _transport of PiT2 [18], we hypothesized that other conserved amino acids in this motif might be critically involved in P_i _transport function of human PiT2 and other members of the PiT family as well. Mutation of human PiT2 H_502 _to alanine created the mutant denoted PiT2 H_502_A. This mutant was analyzed for ^32^P_i _transport function in *X. laevis *oocytes (Figure 3B) and 10A1 MLV and A-MLV receptor functions in CHO K1 cells (Figures 3D-E).

In the experiment shown in Figure 3B, oocytes injected with cRNA encoding human PiT2 supported a ^32^P_i _uptake of 44.96 ±0.46 pmol/oocyte-hour at pH 7.5 in agreement with former studies addressing the Na^32^P_i _uptake function of human PiT2 in *X. laevis *oocytes [18,28,45]. The Pi transport function of human PiT2 H_502_A was severely impaired when compared to that of wildtype PiT2 (*P *= 0.002, 2.36 ±0.56 pmol/oocyte-hour (PiT2 H_502_A)) (Figure 3B).

To analyze whether the human PiT2 H_502_A mutant is properly folded and processed to the cell surface, it was also analyzed for gamma-retroviral receptor function using the transient transfection-infection assay [37]. Eukaryotic expression plasmids encoding human PiT2 and human PiT2 H_502_A mutant protein were transfected into CHO K1 cells non-permissive for infection by A-MLV and 10A1 MLV vector pseudotypes (Figures 3D-E) [28,37]. CHO K1 cells expressing human PiT2 were permissive for infection by both A-MLV and 10A1 MLV vector pseudotypes (Figures 3D-E) in agreement with PiT2's well-described receptor function for A-MLV and 10A1 MLV [8,10,37]. Moreover, the human PiT2 H_502_A mutant supported wildtype PiT2 levels of 10A1 MLV infection (63,940 ±8076 blue cells per dish (PiT2), 50,408 ±4005 blue cells per dish (PiT2 H_502_A), *P *= 0.23) (Figure 3D) and A-MLV infection (13,624 ±862 blue cells per dish (PiT2), 12,235 ±1189 blue cells per dish (PiT2 H_502_A), *P *= 0.48) (Figure 3E). These results demonstrate that the overall membrane topology of human PiT2 H_502_A is preserved, and that the processing of human PiT2 H_502_A to the membrane surface is unaffected by the mutation. Thus, histidine 502 in the 7^th ^TM domain is the second amino acid - besides D_506 _- in the C-terminal PiT family signature sequence [**H**GAN**D**VQNAIGP], which has been shown to be essential for human PiT2 P_i _transport function. While the exact role of the histidine residue in the C-terminal signature sequence still needs to be revealed, its critical role for human PiT2 P_i _transport function emphasizes the importance of the C-terminal PiT family signature sequence in the physiological function of the PiT proteins.

Besides human PiT1 E_70 _and human PiT2 H_502_, six conserved amino acids in human PiT2 and two corresponding positions in human PiT1 have previously been identified as being critical for P_i _transport function [18,27-29]. All these amino acids are located in the ProDom domains (PD001131) suggested in 2004 to define members of the PiT family (Figure 1) [27]. Therefore it is likely that sequences outside these two domains might be dispensable for the P_i _transport function of the PiT proteins, and that a minimal P_i_-transporting unit of the PiT proteins can be identified.

### Alignment of protein sequences of PiT family members from all kingdoms

**Figure 4:**
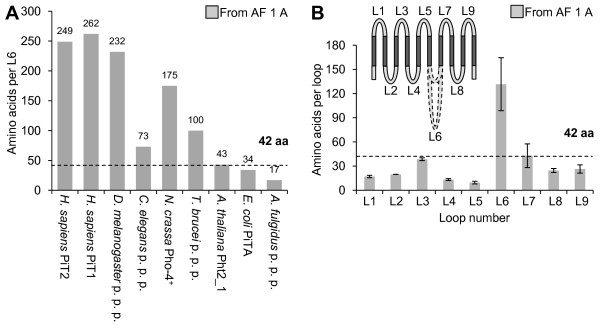
**Investigation of the loop sequence length in PiT family members**. A The amino acid lengths of loop 6 (L6) are plotted for nine PiT family members (*H. sapiens *PiT2, *H. sapiens *PiT1, *N. crassa *Pho-4^+^, *A. thaliana *Pht2_1, *E. coli *PiTA, and putative phosphate permeases from *D. melanogaster*, *C. elegans*, *T. brucei*, and *A. fulgidus*). The L6 lengths are defined by the predicted TM domains in the protein sequences of the PiT family members; see alignment in and legend to Additional File 1 Figure A (AF 1 A). The maximum limit of a loop length (42 amino acids) estimated in Figure 4B is indicated on the figure. It illustrates that loop lengths at 1 to 42 amino acids define a loop sequence and loop lengths at 43 amino acids or higher defines a domain. B The numbers of amino acids in loop 1 (L1) to loop 9 (L9) in the protein sequences listed in the legend to A are shown. The loop lengths were defined by the sequences connecting the predicted TM domains in the protein sequences for the nine PiT family members; see alignment in and legend to Additional File 1 Figure A (AF 1 A). Data are the mean value of (n) numbers of loops counted ±SEM, see Additional File 2 for data. The stippled line indicates the maximum length for a loop sequence (L3) which is ~ 42 amino acids given with 95% confidence (38.6 ±3.4 amino acids ~ 35 to 42 amino acids). Note that the 95% confidence interval for L7 is 42.9 ±28.8 amino acids, illustrating that this loop length is subjected to high uncertainty because of an unusually long L7 in *E. coli *PiTA. The 95% confidence interval for L7 calculated when excluding L7 *E. coli *PiTA is 28.3 ±2.4 amino acids. The topology model indicates the positions of L1 to L9; stippled loops indicate the observed variable lengths of L6 (the large intracellular domain).

A previously published alignment of human PiT1 and human PiT2 protein sequences shows that the L6 loop - the large intracellular domain - is the region where these sequences diverge the most [8] (Additional File 1 Figure A). Moreover, alignment of human PiT1 and *N. crassa *Pho-4^+ ^shows that the large intracellular domain (L6) is smaller in Pho-4^+^, whereas the rest of the Pho-4^+ ^protein sequence aligns well with the protein sequence of human PiT1 [20] (Additional File 1 Figure A). To further address this, we counted the number of amino acids in the large intracellular domain (L6) of nine different PiT family members and plotted the lengths according to their phylogenetic relationship in Figure 4A. The figure shows that PiT family members from archaea and bacteria harbor the shortest L6 loops whereas the PiT-proteins from chordates harbor the longest L6 loops (Figure 4A, see also Additional File 1 Figure A). Note that the L6 loop of the *C. elegans *putative phosphate permease is unexpectedly short (73 amino acids), and according to the plot we would have expected a L6 loop length for this protein in the interval between 175 and 232 amino acids (Figure 4A). The observed differences in the L6 loop lengths of PiT family members from different species thus suggest that the L6 loop evolved from being a regular loop to become a regular domain during evolution. In order to address this issue, we counted the number of amino acids in all loops (L1 to L9) in the nine PiT family members and plotted the average loop lengths ±SEM in Figure 4B. The figure shows that the L6 loop in average is much larger than all other loops (L6: >131.7 ±32.8 amino acids, Figure 4B); see Additional File 2 for data to Figure 4B. The figure also shows that the variation in the L7 loop lengths is substantial (42.9 ±14.7 amino acids), see Figure 4B legend for discussion. Thus, with 95% confidence the longest regular loop is the L3 loop with a maximum length of 42 amino acids, see legend to Figure 4B for discussion. The definition of the maximum length of a loop also has the impact that the L7 of *E. coli *PiTA consisting of 160 amino acids (Additional File 1 Figure A) has to be considered a domain. In summary, analysis of the sizes of the loop sequences L1 to L9 in nine PiT family members from all kingdoms led to the determination of a limit of maximum 42 amino acids in a regular loop sequence - and sequences longer than 42 amino acids are highly likely domains. In support of our calculations of the maximum loop length for PiT-proteins is a previous study of 243 transmembrane domain-containing sequences, with 146 sequences being multi-transmembrane spanning, showing that ~90% of the loops are shorter than 40 amino acid residues [46]. Another study supporting our finding is the analysis of loops in 79 existing 3D structures of transmembrane proteins showing that the majority of loops connecting transmembrane domains are shorter than 50 amino acid residues [47].

The proteins in Figure 4A with L6 loop sizes smaller than 42 amino acids are the archaeal putative phosphate permease and the bacterial PiTA protein, implying that single cell organisms without nuclei that rarely harbor membrane-bound organelles cope without the large intracellular domain, whereas single cell animals (protozoan's) with nuclei and membrane-bound organelles have distinct L6 domains as shown for the *T. brucei *putative phosphate permease (Figure 4A). Altogether this suggest a role(s) for the large intracellular domain, which is not directly related to P_i _transport *per se*, and it also suggest that the large intracellular domain (L6) may have increased in length during the evolution from archaea to chordata as a consequence of adaptation to more complex environments.

**Figure 5:**
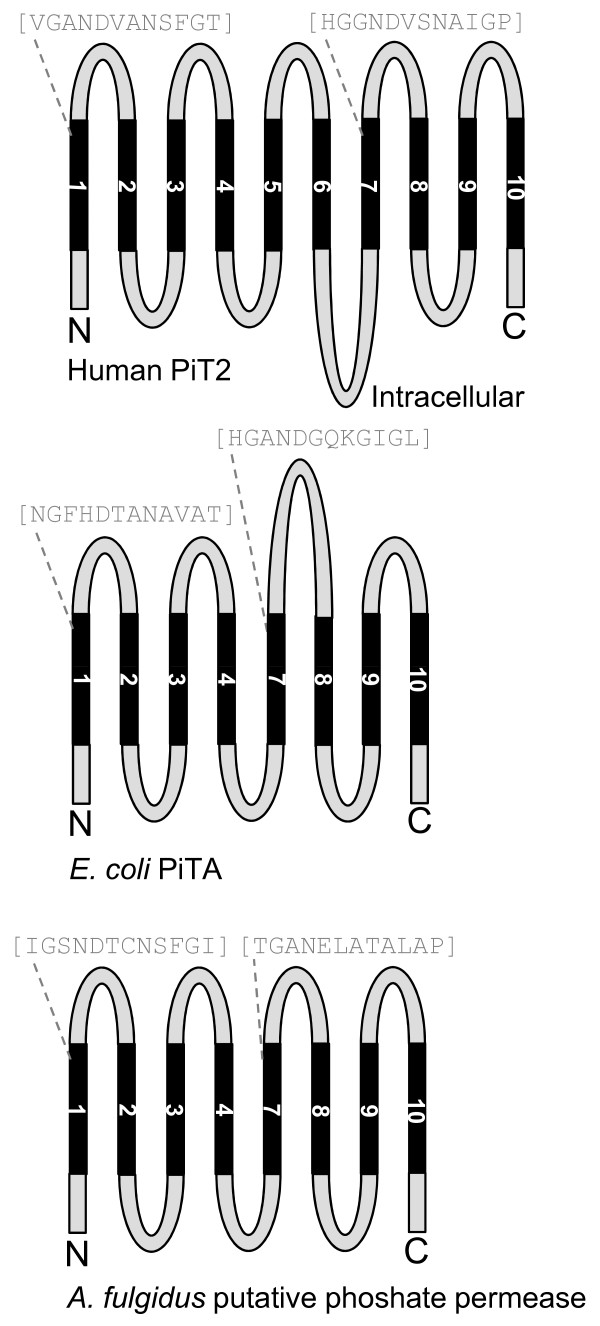
**Predicted topologies of *H. sapiens *PiT2, *E. coli *PiTA, and *A. fulgidus *putative phosphate permease**. Illustrations of the putative topology of *H. sapiens *PiT2, *E. coli *PiTA, and *A. fulgidus *putative phosphate permease are shown. TM domains were predicted using the TMHMM server (*H. sapiens *PiT2) and the DAS server (*E. coli *PiTA and *A. fulgidus *putative phosphate permease) (Additional File 1 Figure A), see "Methods" for description. The N-terminal and C-terminal PiT-family signature sequences [18] are given in grey letters, and grey stippled lines indicate the predicted placement.

Besides a difference in the lengths of L6, a difference in the number of TM domains in the PiT family members was observed (Additional File 1 Figures A and B). The illustration of TM domain conservedness (black boxes) and TM domains, which are suggested by us to be present but not predicted by protein sequence analysis using the TMHMM server (red boxes, see argumentation in legend to Additional File 1 Figure A), shows the following conservedness of TMs: TM 4, TM 8, TM 10 (fully conserved) > TM 5, TM 6 (fully conserved in eukaryotes) > TM 1, TM 2, TM 3 > TM 9 > TM 7 (least conserved) (Additional File 1 Figure B). The most prominent observation is that *E. coli *PiTA and *A. fulgidus *putative phosphate permease both lack the 5^th ^and 6^th ^TM domains (Additional File 1 Figures A and B). This in addition to the previous observation that these two proteins also lack the L6 domain (Figure 4A), suggest that the 5^th ^and 6^th ^TM domains and the L6 domain are dispensable for P_i _transport function, and that a basic P_i_-transporting unit of the PiT family members can be identified. This unit would consist of regions flanking the large intracellular domain (L6) but highly likely also be devoid of the 5^th ^and 6^th ^TM domains. Interestingly, in support of this theory, drawing of the putative topology models for human PiT2, *E. coli *PiTA, and *A. fulgidus *putative phosphate permease based on the alignment in Additional File 1 Figure A, shows that the bacterial and archaeal proteins have a predicted eight TM backbone where the N-terminal PiT-family signature sequence is placed in the 1^st ^extracellular loop (L1) and the C-terminal PiT family signature sequence is placed in the 3^rd ^extracellular loop (L7) (Figure 5). In comparison, the drawing of the putative topology model for human PiT2 shows a backbone of 10 TM domains where the N-terminal and C-terminal PiT-family signature sequences are placed in the 1^st ^extracellular loop (L1) and the 4^th ^extracellular loop (L7), respectively (Figure 5). An interpretation of these drawings could be that the intra-protein locations of the N-terminal and C-terminal PiT-family signature sequences are of importance, and that TM 1 to TM 4 and TM 7 to TM 10 constitute a core sustaining the P_i_-transporting function whereas TM 5 and TM 6 and the large intracellular domain (L6) constitute a regulatory unit. Finally, the amino acids identified as being critical for P_i _transport function are located in the ProDom domains suggested in 2004 (TM 1 to TM 4 and TM 7 to TM 10) [27] (Figure 1) in agreement with the 5^th ^and 6^th ^TM domains and the large intracellular domain (L6) might be dispensable for the P_i _transport function.

### Design of human PiT2 truncation mutants

To identify the minimal P_i_-transporting unit, two human PiT2 truncation mutants were analyzed. They were designed to address the P_i _transport function and the gamma-retroviral receptor functions of: 1) A human PiT2 mutant protein, which consists of the 10 TM domains and a L6 loop of 18 amino acids (human PiT2 P_236_-S_253_) creating the mutant human PiT2ΔR_254_-V_483_. The human PiT2ΔR_254_-V_483 _mutant does not resemble a naturally occurring homolog found in lower species, and it is merely designed to address if the large intracellular domain is dispensable for Na^+^-dependent P_i_-uptake (Figure 1), and 2) A human PiT2 mutant protein that resembles an archaeal and bacterial homolog with respect to protein composition, i.e., lacking the 5^th ^and 6^th ^TM domains and the large intracellular domain (L_183_-V_483_) (human PiT2ΔL_183_-V_483_) (Figure 1). Note that in the Salaün model the 5^th ^and 6^th ^TM domains correspond to TMVI and TMVII (Figure 2).

### The large intracellular domain (R_254_-V_483_) of human PiT2 is dispensable for P_i _transport function whereas the fragment L_183_-V_483 _is more critical for P_i _transport function

**Figure 6:**
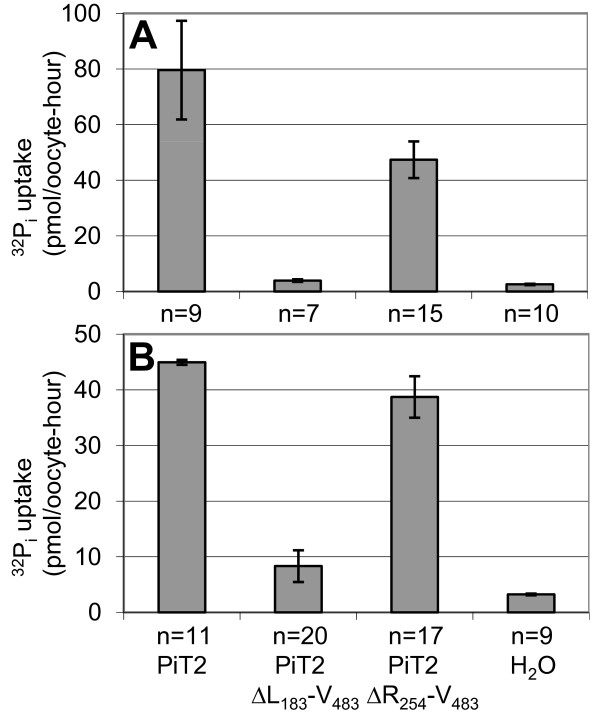
**Na^32^P_i _uptake mediated by human PiT2 and truncation mutants analyzed in *X. laevis *oocytes**. Oocytes were injected with H_2_O or cRNA of the indicated constructs. Two (experiment A) or three (experiment B) days later, a ^32^P_i _uptake assay was performed and the ^32^P_i _uptake in individual oocytes was measured. Data are the mean value of (n) numbers of oocytes ±SEM, see Additional File 2 for data and statistics.

The Na^+^-dependent ^32^P_i _transport function of wildtype human PiT2 and the human PiT2-derived truncation mutants PiT2ΔL_183_-V_483 _and PiT2ΔR_254_-V_483 _(Figure 1) were analyzed in *X. laevis *oocytes (Figure 6).

Oocytes injected with cRNA encoding human PiT2 supported a ^32^P_i _uptake of 79.61 ±17.74 pmol/oocyte-hour (Figure 6A) and 44.96 ±0.46 pmol/oocyte-hour (Figure 6B) at pH 7.5 in agreement with previous results [18,28,45].

The ^32^P_i _transport activities of the PiT2 mutant lacking the major part of the large intracellular domain, human PiT2ΔR_254_-V_483_, (47.38 ±6.59 pmol/oocyte-hour (Figure 6A) and 38.74 ±3.73 pmol/oocyte-hour (Figure 6B)) were indistinguishable from those of PiT2 (*P *= 0.119) (Figure 6A) and *P *= 0.553 (Figure 6B)); see Additional File 2 for data and statistics to Figure 6. Thus, the large intracellular domain of human PiT2 but 18 amino acids (fragment R_254_-V_483_) is dispensable for its P_i _transport function.

The ^32^P_i _transport activity of the human PiT2 mutant lacking the large intracellular domain as well as the 5^th ^and 6^th ^TM domains, PiT2ΔL_183_-V_483 _(Figure 1), was severely impaired (3.93 ±0.44 pmol/oocyte-hour (Figure 6A) and 8.33 ±2.85 pmol/oocyte-hour (Figure 6B)) when compared to the P_i _transport function of wildtype PiT2 (*P *= 0.003 (Figure 6A) and *P *= 0.004 (Figure 6B)). However, interestingly the mutant did support low levels of P_i _uptake significantly different from H_2_O-injected oocytes (2.56 ±0.24 pmol/oocyte-hour (Figure 6A) and 3.24 ±0.17 pmol/oocyte-hour (Figure 6B)) (*P *= 0.011 (Figure 6A) and *P *= 0.008 (Figure 6B)).

### Viral receptor function of mutant PiT2 proteins

Using the transient transfection-infection assay, we analyzed whether the deletions in human PiT2 affected their viral receptor functions for A-MLV and 10A1 MLV. Eukaryotic expression plasmids encoding human PiT2 and the mutant proteins were transfected into CHO K1 cells. As expected, human PiT2 transfected cells were permissive for infection by both 10A1 MLV and A-MLV vector pseudotypes (Table 1). While the human PiT2 truncation mutant lacking the large intracellular domain, human PiT2ΔR_254_-V_483 _(Figure 1) was a fully functional P_i _transporter (Figure 6), it only supported low levels of PiT2 cognate gamma-retroviral infection (Table 1). Note that human PiT2ΔR_254_-V_483 _was tested once for A-MLV receptor function and twice for 10A1 MLV receptor function. The A-MLV study was done in parallel to a 10A1 MLV receptor function study using the same set of plasmid precipitates. Interestingly, the human PiT2 truncation mutant lacking the 5^th ^and 6^th ^TM domains in addition to the large intracellular domain, human PiT2ΔL_183_-V_483 _(Figure 1), supported substantial levels of PiT2 cognate gamma-retroviral infection (Table 1) [31] showing that its low levels of P_i _transport function were not due to incorrect processing of this mutant to the cell surface.

**Table 1:** Levels of 10A1 MLV and A-MLV entry supported by human PiT2 and derived truncation mutants^a^.

Construct^b^	No. (%) of cells infected^c^							
	
	A-MLV			10A1 MLV				
		
	Expt 1^d^	Expt 2^d^	Expt 4	Expt 1^d^	Expt 2^d^	Expt 3^d^	Expt 4	Expt 5
PiT2 (pOJ74)	100 ±22	100 ± 8	100 ±12	100 ± 5	100 ± 8	100 ± 9	100 ±11	100 ± 9
PiT2ΔL_183_-V_483_	25 ± 1	13 ± 2	ND	10 ± 1	12 ± 4	24 ± 3	ND	25 ± 3
PiT2ΔR_254_-V_483_	ND^e^	ND	4 ± >1	ND	ND	ND	1 ± >1	1 ± >1
Empty vector^f^	<0.0008	<0.002	<0.01	<0.002	<0.001	<0.001	<0.007	<0.003

^a^The experimental setup is described in the text. A-MLV and 10A1 MLV vector pseudotypes were tested on the same precipitates made from CsCl-purified plasmids in experiment 4. In experiment 5, Qiagen maxiprep-purified plasmids were used for preparing precipitates and only the 10A1 MLV vector pseudotype was tested.

^b^Receptor and mutant receptor sequences were cloned into pcDNA1A^R^tkpA.

^c^The data are averages of three independent transfections ± SEM. The average number of blue cells per three 60-mm-diameter dishes transfected with a plasmid encoding PiT2 was assigned a value of 100% (57,000, 90,000, and 32,000 blue cells per dish for A-MLV in experiment 4 and for 10A1 MLV in experiments 4 and 5, respectively).

^d^Data are from Bøttger and Pedersen 2004 [31]; please see article for details.

^e^ND, not determined.

^f^Values are based on the detection limit of 1 blue cell per three 60-mm-diameter dishes.

PiT2 regions directly involved in receptor function for 10A1 MLV and A-MLV have also been identified by expression of chimeric proteins in CHO K1 cells and were found to be located in the putative extracellular loops 2 (L3) and 4 (L7) (Figure 1) [26,37,48,49]. Both of the human PiT2 mutants, PiT2ΔR_254_-V_483 _and PiT2ΔL_183_-V_483_, harbor extracellular loops 2 (L3) and 4 (L7) according to the Johann PiT2 model (Figure 1). Based on their - here identified - P_i _transport abilities, it is unlikely that PiT2ΔR_254_-V_483 _is less expressed at the cell surface than PiT2ΔL_183_-V_483_, and the observation that the less truncated human PiT2 mutant protein is a worse gamma-retroviral receptor than a more heavily truncated human PiT2 mutant protein might instead reflect a disturbance of the folding and/or conformation of the extracellular loops 2 (L3) and 4 (L7) due to the sole presence of the extracellular loop 3 (L5) without the large intracellular domain in PiT2ΔR_254_-V_483_.

### Intron-exon borders of the human PiT genes *SLC20A1 *and *SLC20A2*

The human PiT proteins are encoded by genes that localize to different chromosomes. The human gene, *SLC20A1*, encoding the PiT1 protein is located on chromosome 2 at position q13 [50,51], and the human gene, *SLC20A2*, encoding the PiT2 protein is located on chromosome 8 at position p11.2 [8,52,53].

To analyze the gene structure of *SLC20A1 *and *SLC20A2*, the intron-exon borders in each of the genes were determined using the SPIDEY mRNA-to-genome DNA alignment as described in "Methods". The intron-exon borders are marked with stars (✰) and vertical lines in the PiT1 and PiT2 protein sequences in the alignment of nine PiT family members in Additional File 1 Figure A.

Eight out of nine intron-exon borders (labeled ✰ a to e and ✰ g to i on PiT1 and PiT2 in Additional File 1 Figure A) in *SLC20A1 *and *SLC20A2 *are predicted to be homologous. One intron-exon border (labeled ✰ f^1 ^(*SLC20A2*) and f^2 ^(*SLC20A1*)) are displaced giving a gap corresponding to 12 amino acids (~36 nucleotides). These two borders are placed in the middle of the genome sequences, which encode the large intracellular domain (L6) of the human PiT proteins. As seen from Additional File 1 Figure A, the alignment between the human PiT proteins in this region is poor and the gap highly likely reflects this, and not a significant difference in intron-exon structure between *SLC20A1 *and *SLC20A2*.

Interestingly, in support of the theory that the 5^th ^and 6^th ^TM domains can be dispensable for P_i _transport function, is the observation that these TM domains are encoded by two different exons, see Additional File 1 Figure A (✰ labeled c to d, and ✰ labeled d to e), and therefore the possibility exists that the sequences in these two exons have entered later in evolution.

### Specialized functions of the mammalian PiT proteins

Mammalian PiT proteins are expressed in all tissues investigated and due to their broad expression profiles, they have been suggested to accommodate house-keeping functions, i.e., supplying cells with P_i _to maintain basic cellular functions [2,20,54]. However, in recent years additional specialized functions of the PiT proteins have been reported. These include roles for PiT2 in proximal tubule phosphate reabsorption [55], and for PiT1 in regulation of parathyroid gland PTH production [56,57], cell proliferation [29,58,59], and in tumor necrosis factor (TNF) induced apoptosis [60]. Recent studies also indicate that both the PiT proteins function as P_i _sensors [27,56], reviewed in [61]. Interestingly, some of these functions, that is, PiT2's suggested role in P_i _sensing [27] and PiT1's role in cell proliferation and TNF-induced apoptosis [29,59,60] have been shown to be independent of the P_i _transport functions of the proteins.

PiT1 has also been implicated in normal chondroblastic and osteoblastic differentiation and mineralization processes [62-66], as well as trans-differentiation of vascular smooth muscle cells to cells with characteristics of chondro-/osteoblasts in the pathologic process of vascular calcification at hyperphosphatemia [67]. More rodent *in vivo *models have been used to study the role of PiT1 in normal bone formation and/or embryonic development. Rats with transgenic overexpression of PiT1 showed no major bone deformity during skeletal development [57]. However, these rats displayed a slight but significant decrease in the bone mineral content of the whole skeleton together with a reduction albeit non-significant in the total bone area [57]. The role of PiT1 during embryonic mouse development has been studied by two different groups employing early conditional excision of *SLC20A1 *Exons 3-4 [68] and *SLC20A1 *Exon 5 [59], which resulted in homozygous embryonic lethality. Both studies find that the embryos are anemic and do not survive past E12.5, at which stage the morphology shows reduced growth [59,68]; the anemia was found to be due to severe defects in liver development [59]. Comparison of wildtype mice to mice with low (15%) expression of PiT1 mRNA showed that some of the latter mice displayed impaired bone mineralization at birth, while 15-days old mice showed no major differences in mineralization [59]. Interestingly, in embryos (E11.5) lacking

PiT1 expression Beck and coworkers found an upregulated PiT2 expression, which however could not rescue the embryos past E12.5, and the authors therefore suggest that the critical non-redundant role of PiT1 in development is not P_i_-uptake [59]. Altogether, the *in vivo *studies do not exclude a role for PiT1 in normal bone formation, although they imply that PiT1 is not critical for the early skeletal developmental processes.

The alignment and analyses of exon structure together with the observed P_i _transport functions of the PiT2 deletion mutants presented here might suggest that the regions of the PiT proteins involved in the P_i_-transport independent functions map to sequences in the 5^th ^and 6^th ^TM domains and/or in the large intracellular domain. In line with this, we are currently investigating the function of the large intracellular domain of the human PiT2 protein and our results support the hypothesis that the large intracellular domain has other functions than P_i _transport.

## Conclusions

Investigation of the P_i _transport and retroviral receptor functions of the human PiT proteins has allowed for identification of a histidine residue (human PiT2 H_502_) in the C-terminal PiT family signature sequence as being critically involved in P_i _transport function. Moreover, we show that a PiT1 glutamate residue (human PiT1 E_70_) positioned in the 2^nd ^TM domain is critical for P_i _transport function in agreement with the former identification of the equivalent glutamate in human PiT2 (human PiT2 E_55_) as being critical for P_i _transport function [28].

We have shown that a human PiT2 mutant consisting of the 10 TM domains and minor loops (human PiT2ΔR_254_-V_483_) transports P_i _as wildtype PiT2, proving that the large intracellular domain (L6) is dispensable for P_i _transport function. A further truncated human PiT2 mutant consisting of the 1^st ^to 4^th ^TM domains linked to the 7^th ^to 10^th ^TM domains and the minor loop sequences connecting the TMs (human PiT2ΔL_183_-V_483_), and which resembles archaeal and bacterial homologs, sustained low levels of P_i _transport. This protein harbors the ProDom domains defining the PiT family members and, moreover, harbors all the amino acids so far identified as being critical for P_i _transport function.

The above results showing that truncated human PiT2 mutant proteins - one of which resembles a phosphate permease from bacteria and a putative phosphate permease from archaea - support P_i _transport, point to the conclusion that the overall structure of the PiT family proteins has remained unchanged during evolution and that a basic P_i_-transporting unit exists.

## List of abbreviations used

10A1 MLV: retrovirus closely related to A-MLV, A-MLV: amphotropic murine leukemia virus, CHO K**1: **Chinese hamster ovary K1, GALV: gibbon ape leukemia virus, FeLV-B: feline leukemia virus subgroup B, P_i_: inorganic phosphate, PiT: P_i _transporter, TM: transmembrane.

## Authors' contributions

PB and LP conceived the study and designed the experiments. PB did the experimental work, and drafted the manuscript. PB and LP edited and approved the final version of the manuscript.

## Supplementary Material

Additional file 1 **Protein sequence alignment of nine PiT family members from all kingdoms**. A The 10 putative TM domains according to the Johann topology model are shown on the human PiT2 sequence using black boxes with white filling [8,20]; the putative large intracellular domain (L6) of human PiT2, according to this model, spans the amino acid sequence: P_236_-V_483_. The N-terminal and C-terminal PiT family signature sequences [18] are shown on the alignment in black boxes with grey filling. Human PiT1 E_70 _in the 2^nd ^TM domain and human PiT2 H_502 _in the 7^th ^TM domain are indicated with circles. The TMHMM-predicted TM domains of the eukaryotic protein sequences for PiT family members and the DAS-predicted TM domains of the prokaryotic protein sequences for PiT family members are shown in black bold. The red bold sequences represent TM-domains, which we suggest exist, however, they were not predicted by the servers: *N. crassa *Pho-4^+ ^TM 1 (sequence Q_5_-I_24_) is suggested to be homologous to the TM 1 predicted in the *C. elegans *putative phosphate permease protein sequence. The presence of Pho-4^+ ^TM 1 is also based on the assumption that the N-terminal PiT-family signature sequences should be placed equivalently (extracellularly in L1) in all PiT family members. *A. thaliana *Pht2_1 TM 2 (sequence A_187_-G_211_) is suggested to be homologous to the TM 2 predicted in the *T. brucei *putative phosphate permease protein sequence. The presence of Pht2_1 TM 2 is also based on experimental assignment of the L6 for rat PiT2 to the cytoplasmic space [21], and Pht2_1 therefore requires a TM 2 to fulfill this criteria. *H. sapiens *PiT2 TM 3 (sequence T_83_-A_105_) is suggested to be homologous to the TM 3 predicted in the *H. sapiens *PiT1 protein sequence. Investigation of a human PiT1/PiT2 chimera where the PiT1 backbone harbors the human PiT2 sequence G_120_-V_141 _showed that this sequence conferred A-MLV receptor function upon human PiT1 [48], and the G_120_-V_141 _sequence is therefore highly likely extracellular in both human PiT paralogs and this requires the presence of TM 3 in human PiT2. TM 7 domains in putative phosphate permeases from *C. elegans *(sequence Q_330_-A_349_), *D. melanogaster *(sequence M_472_-G_491_), *T. brucei *(sequence Y_346_-A_365_), and *N. crassa *Pho-4^+ ^(sequence Y_318_-A_337_) are suggested to be homologous to the TM 7 predicted in *H. sapiens *PiT2 and PiT1 sequences. The presence of TM 7 in putative phosphate permeases from *C. elegans*, *D. melanogaster*, *T. brucei*, and *N. crassa *Pho-4^+ ^is also based on the assumption that the C-terminal PiT-family signature sequences should be placed equivalently (extracellularly in L7) in all PiT family members. Moreover, investigation of a Pho-4^+^/human PiT2 chimera where the Pho-4^+ ^backbone harbors the human PiT2 sequences C_117_-I_143 _(stretch in L3) and L_512_-A_531 _(stretch in L7) showed that these sequences confer A-MLV receptor function upon Pho-4^+^[26], and these sequences are therefore highly likely extracellular and this requires the presence of a TM 7. Similarly, investigation of a Pho-4^+^/human PiT1 chimera where the Pho-4^+ ^backbone harbors the human PiT1 sequence L_545_-S_556 _(stretch in L7) showed that these sequences confer GALV receptor function upon Pho-4^+^[25]. *H. sapiens *PiT2 TM 9 (sequence G_571_-S_593_) and *H. sapiens *PiT1 TM 9 (sequence G_599_-S_521_) are suggested to be homologous to the TM 9 predicted in RPHO-1 *R. norvegicus *(human PiT1 ortholog) protein sequence G_601_-S_623 _[Swiss-Prot:Q9JJP0] using the TMHMM server (data not shown). *N. crassa *Pho-4^+ ^TM 9 (sequence L_523_-G_545_) is suggested to be homologous to the TM 9 predicted in *C. elegans *putative phosphate permease protein sequence. The TM 9 is required to orient the TM 10 equivalently in all PiT family members. Lower case letters represent TMHMM- or DAS-predicted TM sequences, which we based on either too small length to comprise a TM or due to suggested extracellular position (see above) found were non-compatible with regular TM domains; however, these sequences might instead "dip" into the membrane lipid bilayer. It should be noted that these sequences are counted as being part of the loop sequences in Figure 4. A star (✰) (labeled a to i) and a vertical line indicate the position of an intron-exon border in each of the human PiT genes determined by use of the SPIDEY mRNA-to-genome DNA alignment as described in "Methods". Below the alignment, the names, species, phylas, kingdoms, Swiss-Prot accession numbers, and the amino acid lengths of the nine proteins are given. B The server-predicted TM domains (black boxes) and the by us suggested TM domains (red boxes) for each of the nine PiT family members are depicted in order to illustrate the conservedness of the TM domains: TM 4, TM 8, TM 10 (fully conserved) > TM 5, TM 6 (fully conserved in eukaryotes) > TM 1, TM 2, TM 3 > TM 9 > TM 7 (least conserved). The white asterisk indicates a prediction of a unique TM domain in the unusually long N-terminal sequence of *A. thaliana *Pht2_1. Click here for file

Additional File 2 **Data and statistics**. Average ^32^P_i _uptakes in oocytes given as pmol/oocyte-hour ±SEM, information regarding the number (n) of oocytes measured, and the statistics (*P *values) for Figures 3A-B and Figure 6 are available in Additional File 2. Average numbers of blue (infected) cells per dish from three dishes ±SEM and the statistics (*P *values) for Figures 3C-E are available in Additional File 2. Average loop lengths given as amino acids ±SEM and information regarding the number (n) of loops counted for Figure 4B are available in Additional File 2. Click here for file
