# Supplementary material for: Mapping of the minimal inorganic phosphate transporting unit of human PiT2 suggests a structure universal to PiT-related proteins from all kingdoms of life
Source: BMC Biochem. 2011 May 17;12:21. doi: 10.1186/1471-2091-12-21 (PMC3126765; doi:10.1186/1471-2091-12-21)

A

```
1. PiT2              ------------------------------------------------------------
2. PiT1              ------------------------------------------------------------
3. C. elegans        ------------------------------------------------------------
4. D. melanogaster   ------------------------------------------------------------
5. Pho-4+            ------------------------------------------------------------
6. T. brucei         ------------------------------------------------------------
7. A. fulgidus       ------------------------------------------------------------
8. Pht2_1            MTLPYRFSSVRNHSLLLKTSHLCTPRSALGCCFSPKESPFFRKNTAQFLSPQKHTSLPLK 60
9. PiTA              ------------------------------------------------------------


PiT2                 ------------------------------------------------------------
PiT1                 ------------------------------------------------------------
C. elegans           ------------------------------------------------------------
D. melanogaster      ------------------------------------------------------------
Pho-4+               ------------------------------------------------------------
T. brucei            ------------------------------------------------------------
A. fulgidus          ------------------------------------------------------------
Pht2_1               LVCPLASFSSYADSEGEEQHHADQPIQNPHESSTVSNESDGKGNAEATGDFSGMAQAFHI 120
PiTA                 ------------------------------------------------------------

                                                         TM 1
PiT2                 ----------------------------MAMDEYLWMVILGFIIAFILAFSVGANDVANS 32
PiT1                 -------------MATLITSTTAATAASGPLVDYLWMLILGFIIAFVLAFSVGANDVANS 47
C. elegans           -------MDTATAMLSGASSTFAGVVGDFDQSSVLWALIVGIILAFLLGAGMGANDVSNA 53
D. melanogaster      --------------------------MESFAPELLWMVVIGFLIAFVLAFGIGANDVANS 34
Pho-4+               ----------------------------MVLHQFDYLLAIGTIFAALDAWNIGANDVANS 32
T. brucei            -----------------------------MVHPYLWVIIVGGIVSFLTGCGVGMNDLANS 31
A. fulgidus          -----------------------------------MDVTIALVAALAIAFAIGSNDTCNS 25
Pht2_1               SSTTARAISIVIAFSALTLPIFMKSLGQGLALKTKLLSYATLLFGFYMAWNIGANDVANA 180
PiTA                 ----------------------MLHLFAGLDLHTGLLLLLALAFVLFYEAINGFHDTANA 38

                        L1            TM 2                 L2
PiT2                 FGTAVGSGVVTLRQACILASIFETTGSVLLGAKVGETIRKGIIDVNLYNETVETLMAGEV 92
PiT1                 FGTAVGSGVVTLKQACILASIFETVGSVLLGAKVSETIRKGLIDVEMYNSTQGLLMAGSV 107
C. elegans           FGTSVGSGVLTIIQAYILASIFETLGSVLVGWSVVDTMRKGVVDISQYENTPKEFLLGQI 113
D. melanogaster      FGTSVGSGVLTIRQACVLATICEISGAVLIGYKVSDTMRKGILEVGLYEGAEEVLMLGCV 94
Pho-4+               WATSVAARSVTYLQAMILGSIMEFAGSVGVGARVADTIRTKVVDTTLFADDPALLMLGMV 92
T. brucei            FGTTYGSRVLNLWQIVILASICELVGAVSLGSEVTSTISGGIANPMTFANEPYVLMYGMM 91
A. fulgidus          FGICVGCGLLTMKRAAYILFLLVFAGMLFGSGRIMDTVGREMVELNEIVVSVSLIIS--- 82
Pht2_1               MGTSVGSGALTIRQAVMTAAVLEFSGALLMGTHVTSTMQKGILMANVFQGKDMLLFAGLL 240
PiTA                 VATVIYTRAMRSQLAVVMAAVFNFLGVLLGGLSVAYAIVHMLPTDLLLNMGSSHGLAMVF 98

                        a
                        ☆ TM 3                           L3
PiT2                 SAMVGSAVWQLIASFLRlpisgthcivgstigfslvaigtKGVQWME----------LVK 142
PiT1                 SAMFGSAVWQLVASFLKLPISGTHCIVGATIGFSLVAKGQEGVKWSE----------LIK 157
C. elegans           AVLGGCSAWLLIATVLHMPVSTTHSLVGATIGFSVVLRGFNGIQWMA----------IVK 163
D. melanogaster      AALASSAVWLLVATFLKLPISGTHSIVGSTIGFSLVARGVQGLKWST----------LGT 144
Pho-4+               CAVVASSIYLTMATRFGLPVSTTHSIMggvigmgiaavgadgvqwvc----ssiNDGVVS 148
T. brucei            CALSATFIWLLFATMMSLPVSSThsiagaiigfalvyggfgavsfaKKIDEFPYVTGVAP 151
A. fulgidus          ------SAAIVAANYLRTPVSSHQAIVmsligsAFALGNRIDVSTVA------------K 124
Pht2_1               SSLAAAGTWLQVASYYGWPVSTTHcivgsmvgfglvyggagavfwss----------LAK 290
PiTA                 SMLLAAIIWNLGTWYFGLPASSSHtligaiigigltnALMTGTSVVDALN----IPKVLS 154
```

```
                      b           TM 4                 c L4          TM5
PiT2                  IVASWFISPLLSGFMSGLLFVLIRIFIL---KKEDPVPNGLRALPVFYAATIAINVFSIM 199
PiT1                  IVMSWFVSPLLSGIMSGILFFLVRAFIL---HKADPVPNGLRALPVFYACTVGINLFSIM 214
C. elegans            IVCSWFISPVLSGIISSIIYMIVDHTVL---RTANPLKNGLRALPVFYFVCMAFNALMVF 220
D. melanogaster       IVGSWFISPVLSGIVSILLFLAIRRFIL---RAQEPLKAGFRSLPIFYGVTFFINVISVV 201
Pho-4+                VFLAWVIAPGLAGAFASIIFLVTKYGVL---LRSNPVYKAFVMVPIYFGITAALLCMLLL 205
T. brucei             IIASWFISPVFAGAVAASLYALLRLVVL---RPANSVNRALFALPLIVGVTFFLESFFVL 208
A. fulgidus           IVLSWVISPFGALLLSIVFYWIMERTLA---KLPALR----------------------- 158
Pht2_1                VASSWVISPILGALVSFLVYKCIRRFVYSAPNPGQAAAAAAP---------------VAV 335
PiTA                  IFGSLIVSPIVGLVFAGGLIFLLRRYW-SGTKK--------------------------- 186

                         d  L5          TM 6            L6  e
PiT2                  YTGAPVLGL-VLPMWAIALISFGVALLFAFFVWLFVCPWMRRKITGKLQKEGALSRVSDE 258
PiT1                  YTGAPLLGFDKLPLWGTILISVGCAVFCALIVWFFVCPRMKRKIEREIKCSPSESPLMEK 274
C. elegans            WDGSKLLHFDEIPVWGIVIIVLGVGTIAAAFANFVMKPRIRAKIQ--------------- 265
D. melanogaster       LDGPKLLYMDNIPTWIALTASFGLSLLVALLTQLVVVPLQRRKIAKRLRAENPVKFN--- 258
Pho-4+                WKGG--SYKVTLTNPEIAGTIIGVGAAWALLVTIFLMPWLYRIVILEDWQLRFWHIPLGP 263
T. brucei             FKGA—DSHLHWGPAKASWVAALIGLGAASISAACIPLL---------------------R 246
A. fulgidus           ------------------------------------------------------------ 158
Pht2_1                FVGVASISSAALPLSKIFPIALSQALACGVAGAIVFDRIIRKQLG--------------- 380
PiTA                  ------------------------------------------------------------ 186

PiT2                  --SLSKVQEAESPVFKELPGAKANDDS---TIPLTGAAGET-----LGTSEGTSAGSHPR 308
PiT1                  KNSLKEDHEETKLSVGDIENKHPVSEVGPATVPLQAVVEERTVSFKLGDLEEAPERERLP 334
C. elegans            ----DSEAPPTPPMFSDVESARG-----------------------SAELEEFTEGS--- 295
D. melanogaster       ----FEDSVESSPSGSPKKQRRPLSLVS-EGKPLPAIAEIT----ELVSLSDNSPRTFKL 309
Pho-4+                LLLRRGEVPPPPADGSGVVQDFYAGRLTKEQLAARRAAQNGDSEMAAGAVTSSTSNPSAP 323
T. brucei             RRVR--------------------------LITERAERERAETGMNTAPEISGDAGAISE 280
A. fulgidus           ------------------------------------------------------------ 158
Pht2_1                ------------------------------------------------------------ 380
PiTA                  ------------------------------------------------------------ 186

                        f1          f2
PiT2                  AAYGRALSMTHGSVK--SPISNG-----TFGFDGHTRSDGH-VYHTVHKD-SGLYKDLLH 359
PiT1                  SVDLKEETSIDSTVNGAVQLPNGNLVQFSQAVSNQINSSGHSQYHTVHKD-SGLYKELLH 393
C. elegans            -------------------------------VTNQQK-----TMSTPGKI-RKFFTWLLP 318
D. melanogaster       APFGLAAKNNNALG-------------EEYKIDPQLIKKAEDLLGKASLDNTDLTITSLN 356
Pho-4+                TDGEKGATITKDDSS------------YSHDHSEPAQAAQPQIKTMVGPRPAGPWHSGAV 371
T. brucei             NAAGVGAAVEG--------------------PVDTANRIVPPSSEPTSDSPTTEYQS--- 317
A. fulgidus           ------------------------------------------------------------ 158
Pht2_1                ---------------------------------HLLAKTKSPETSQNQPKTIGFLSDIAG 407
PiTA                  ------------------------------------------------------------ 186

PiT2                  KIHIDRGPEEKPAQESNYRLLRRNNSYTCYTAAICGLPVHATFRAADSSAP-EDSEKLVG 418
PiT1                  KLHLAKVG--DCMGDSGDKPLRRNNSYTSYTMAICGMPLDS-FRAKEGEQKGEEMEKLTW 450
C. elegans            ------------------------------------------------------------ 318
D. melanogaster       FIDEQQQQ---QQQQQNGRKLQECFKRMQSPKEEQKSKTNSIGTDLETGSTKATNNNLQV 413
Pho-4+                LFWYVKWA------------------------------------------------LFRG 383
T. brucei             ------------------------------------------------------------ 317
A. fulgidus           ------------------------------------------------------------ 158
Pht2_1                PTG--------------------------------------------------------- 410
PiTA                  ------------------------------------------------------------ 186
```

```
PiT2              DTVSYSKKRLRYDSYSSYCNAVAEAEIEAEEGGVEMKLASELADPDQPREDPAEEEKEEK 478
PiT1              PNAD-SKKRIRMDSYTSYCNAVSDLHSASE---IDMSVKAAMGLGDRKGSNGSLEEWYDQ 506
C. elegans        -------DRTRSDSRS-------------------------------------------- 327
D. melanogaster   VESGGSLDLMISSTLSPNSSKVPLIESKEALNEQEEELKRTTGGGRRTSG--------AE 465
Pho-4+            VDQDVLSSQQEKSVISSDVEELHAHATHYD------------------------------ 413
T. brucei         ------KNMSRLSMTGVVDEALKFDVQIYD------------------------------ 341
A. fulgidus       ------------------------------------------------------------ 158
Pht2_1            -----------------------------------------------------------T 411
PiTA              ----------------------------------------------RARIHLTPAEREKK 200

                             TM 7            g                  L7
PiT2              DAPEVHLLFHFLQVLTACFG-SFAHGGNDVSNAIGPLVALWL------------------ 519
PiT1              DKPEVSLLFQFLQILTACFG-SFAHGGNDVSNAIGPLVALYL------------------ 547
C. elegans        ----ITQLFSTIQVFTACFA-GFAHGANDVSNAVAPLAAIIS------------------ 364
D. melanogaster   ETPEISMLFSFLQILTATFG-SFAHGGNDVSNAIGPLIALYM------------------ 506
Pho-4+            --NKTEYMYSFLQIMTAAAA-SFTHGANDIANAIGPYATVFQ------------------ 452
T. brucei         --ERVEYVFRYLQVFTAVCA-SFAHGANDVSNAIAPFSAMYS------------------ 380
A. fulgidus       -----VERVLRVLLFIGATVIGFNTGANELATAlapivmfgvmN---------------- 197
Pht2_1            QLEIVYGIFGYMQVLSACFM-SFAHGGNDVSNAigplaaals------------------ 452
PiTA              DGKKKPPFWTRIALILSAIGVAFSHGANDGQKGiglvmlvligvapagfVVNMNATGYEI 260

PiT2              ------------------------------------------------------------ 519
PiT1              ------------------------------------------------------------ 547
C. elegans        ------------------------------------------------------------ 364
D. melanogaster   ------------------------------------------------------------ 506
Pho-4+            ------------------------------------------------------------ 452
T. brucei         ------------------------------------------------------------ 380
A. fulgidus       ------------------------------------------------------------ 197
Pht2_1            ------------------------------------------------------------ 452
PiTA              TRTRDAINNVEAYFEQHPALLKQATGADQLVPAPEAGATQPAEFHCHPSNTINALNRLKG 320

PiT2              ---------------------------------IYKQGGVTQE--AA------------- 531
PiT1              ---------------------------------VYDTGDVSSK--VA------------- 559
C. elegans        ---------------------------------VYRTKSVEQK--EA------------- 376
D. melanogaster   ---------------------------------IYREGSVMQQ--AE------------- 518
Pho-4+            ---------------------------------LWKDGALPEKGKAD------------- 466
T. brucei         ---------------------------------IYINQQVVEEN--D------------- 392
A. fulgidus       ------------------------------------------------------------ 197
Pht2_1            ---------------------------------ilqngaaaggaeiv------------- 466
PiTA              MLTTDVESYDKLSLDQRSQMRRIMLCVSDTIDKVVKMPGVSADD—QRLLKKLKSDMLSTI 379

                          TM 8             L8          h   TM 9
PiT2              --TPVWLLFYGGVGICTGLWVWGRRVIQTMGKDLTP--ITPSSGFTIELASAFTVVIASN 587
PiT1              --TPIWLLLYGGVGICVGLWVWGRRVIQTMGKDLTP--ITPSSGFSIELASALTVVIASN 615
C. elegans        --VPIYVLLYGVLAICIGLWTFGHYVIKTVGTKMSE--INPASGFTIEFGAAMTSLVASK 432
D. melanogaster   --SPIYILIYGGIGISVGLWLWGRRVIETIGNDLTK--ITSSTGFTIEVGAAITVLLASK 574
Pho-4+            --VPVWILVFGASCLVIGLWTYGYNIMRNLGNRITL--QSPSRGFSMELGSAVTVILATR 522
T. brucei         --VPLWILVLGGAGLVVGLATLGVRIMRLLGERITK--ITPSRGFSAELSAALVVSLCSA 448
A. fulgidus       ---VFEAALLGSAMLFLGAWIVSVRVAEVVGKGITA--LDPFTGFAAQFAAGITVLLFTL 252
Pht2_1            --IPMDVLAWGGFGIVAGLTMWGYRVIATIGKKITE--LTPTRGFAAEFAAASVVLFASK 522
PiTA              EYAPVWIIMAVALALGIGTMIGWRRVATTIGEKIGKKGMTYAQGMSAQMTAAVSIGLASY 439
```

```
                          i
                          ☆                 L9
PiT2            IGLPVSTTHCKVGSVVAVGWIRS-------------------------RKAVDWRLFRNI 622
PiT1            IGLPISTTHCKVGSVVSVGWLRS-------------------------KKAVDWRLFRNI 650
C. elegans      LGLPISTTHCLVGSVVAVGALRS-------------------------EDGVKWSTFRNI 467
D. melanogaster IGLPISTTHCKVGSVVFVGHVSASGRKKKSQPNDQTDKETHNEVAPMDDGSVDWHLFRNI 634
Pho-4+          LKLPVSTTQCITGATVGVGLCSGT------------------------WRTINWRLVAWI 558
T. brucei       FGIPVSSTHCITGAVVAISIMDCG------------------------FRKVRWMMVGKM 484
A. fulgidus     IGMPVSTTYCTVGAVTGVGLYKS-------------------------VRGVKFAFLKRI 287
Pht2_1          LGLPISATHTLVGAVMGVGFARG-------------------------LNSVRAETVREI 557
PiTA            TGMPVSTTHVLSSSVAGTMVVDG--------------------------GGLQRKTVTSI 473

                     TM 10
PiT2            FVAWFVTVPVAGLFSAAVMALLMYGILPYV--- 652
PiT1            FMAWFVTVPISGVISAAIMAIFRYVILRM---- 679
C. elegans      FMSWVITLPVSGLISAGIMLIIKWAAL------ 494
D. melanogaster AYAWIVTVPVTALLSAGMMYVLCAIAVDDMGGA 667
Pho-4+          YMGWFITLPVAGIISGCLMGIIINAPRWGYSG- 590
T. brucei       YLGWIFTLLITAAISALLFAQGIYAPSLTSQ-- 515
A. fulgidus     VASWILTPFTAFTLSFILTLLLSQQAF------ 314
Pht2_1          VASWLVTIPVGATLAVIYTWIFTKILSFVL--- 587
PiTA            LMAWVFTLPAAVLLSGGLYWLSLQFL------- 499
```

	Name	Species	Phyla	Kingdom /Domain	Swiss-Prot	Length
1.	PiT2	*H. sapiens*	Chordata	Animalia/Eucaria	Q08357	652 aa
2.	PiT1	*H. sapiens*	Chordata	Animalia/Eucaria	Q08344	679 aa
3.	p.p.p.	*C. elegans*	Nematoda	Animalia/Eucarya	Q17455	494 aa
4.	p.p.p.	*D. melanogaster*	Arthropoda	Animalia/Eucarya	Q9VTG0	667 aa
5.	Pho-4+	*N. crassa*	Ascomycota	Fungi /Eucarya	P15710	590 aa
6.	p.p.p.	*T. brucei*	Euglenozoa	Protista/Eucarya	Q9N930	515 aa
7.	p.p.p.	*A. fulgidus*	Archaeoglobus	Monera /Archaea	O29467	314 aa
8.	Pht2_1	*A. thaliana*	Streptophyta	Plantae /Eucarya	Q38954	587 aa
9.	PiTA	*E. coli*	Proteobacteria	Monera /Bacteria	P37308	499 aa

p.p.p.: putative phosphate permease

B

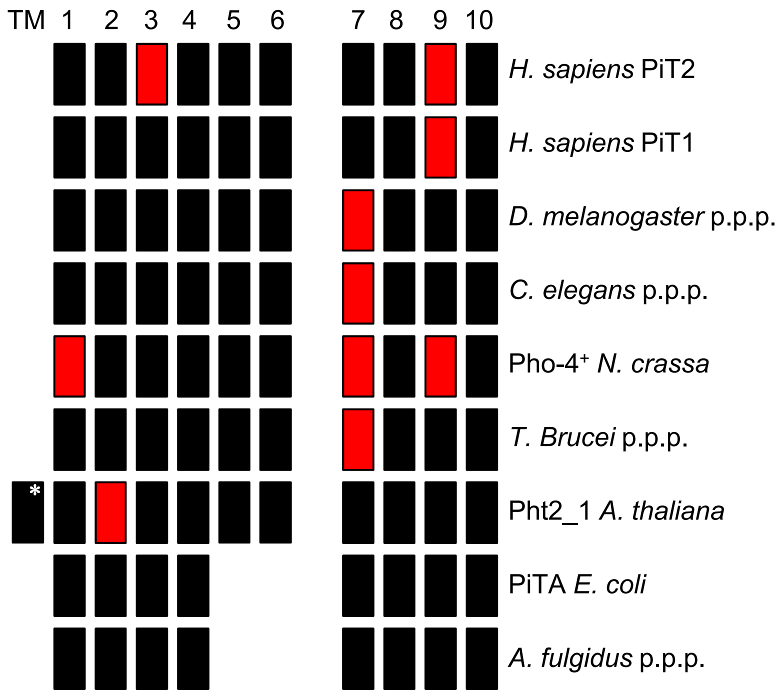

Supplement: Additional file 1 — Protein sequence alignment of nine PiT family members from all kingdoms. A The 10 putative TM domains according to the Johann topology model are shown on the human PiT2 sequence using black boxes with white filling [8,20]; the putative large intracellular domain (L6) of human PiT2, according to this model, spans the amino acid sequence: P236-V483. The N-terminal and C-terminal PiT family signature sequences [18] are shown on the alignment in black boxes with grey filling. Human PiT1 E70 in the 2nd TM domain and human PiT2 H502 in the 7th TM domain are indicated with circles. The TMHMM-predicted TM domains of the eukaryotic protein sequences for PiT family members and the DAS-predicted TM domains of the prokaryotic protein sequences for PiT family members are shown in black bold. The red bold sequences represent TM-domains, which we suggest exist, however, they were not predicted by the servers: N. crassa Pho-4+ TM 1 (sequence Q5-I24) is suggested to be homologous to the TM 1 predicted in the C. elegans putative phosphate permease protein sequence. The presence of Pho-4+ TM 1 is also based on the assumption that the N-terminal PiT-family signature sequences should be placed equivalently (extracellularly in L1) in all PiT family members. A. thaliana Pht2_1 TM 2 (sequence A187-G211) is suggested to be homologous to the TM 2 predicted in the T. brucei putative phosphate permease protein sequence. The presence of Pht2_1 TM 2 is also based on experimental assignment of the L6 for rat PiT2 to the cytoplasmic space [21], and Pht2_1 therefore requires a TM 2 to fulfill this criteria. H. sapiens PiT2 TM 3 (sequence T83-A105) is suggested to be homologous to the TM 3 predicted in the H. sapiens PiT1 protein sequence. Investigation of a human PiT1/PiT2 chimera where the PiT1 backbone harbors the human PiT2 sequence G120-V141 showed that this sequence conferred A-MLV receptor function upon human PiT1 [48], and the G120-V141 sequence is therefore highly likely extracellular in bo [file 1471-2091-12-21-S1.PDF]
