# Supplementary material for: Mapping of the minimal inorganic phosphate transporting unit of human PiT2 suggests a structure universal to PiT-related proteins from all kingdoms of life
Source: BMC Biochem. 2011 May 17;12:21. doi: 10.1186/1471-2091-12-21 (PMC3126765; doi:10.1186/1471-2091-12-21)
Supplement: Additional File 2 — Data and statistics. Average 32Pi uptakes in oocytes given as pmol/oocyte-hour ±SEM, information regarding the number (n) of oocytes measured, and the statistics (P values) for Figures 3A-B and Figure 6 are available in Additional File 2. Average numbers of blue (infected) cells per dish from three dishes ±SEM and the statistics (P values) for Figures 3C-E are available in Additional File 2. Average loop lengths given as amino acids ±SEM and information regarding the number (n) of loops counted for Figure 4B are available in Additional File 2. [file 1471-2091-12-21-S2.PDF]

Figure 3

Analysis of human PiT1 $E_{70}K$ and PiT2 $H_{502}A$ for $Na^{32}P_i$ uptake and gamma-retroviral receptor function.

A-B $^{32}P_i$ uptake in individual oocytes was measured and data are the mean value of (n) numbers of oocytes ±SEM. PiT1 n=11 (A) and n=19 (B), PiT1 $E_{70}K$ n=10 (A), PiT2 n=11 (B), PiT2 $H_{502}A$ n=11 (B), H_2O n=12 (A) and n=9 (B). The *P* values for comparison to PiT1 are as follows: PiT1 $E_{70}K$ *P*=0.002, H_2O *P*=0.01. For comparison to H_2O: PiT1 $E_{70}K$ *P*=0.02. The *P* values for comparison to PiT2 are as follows: PiT2 $H_{502}A$ *P*=0.002, H_2O *P*=0.002. For comparison to H_2O: PiT2 $H_{502}A$ *P*=0.1. The average $^{32}P_i$ uptakes were 119.86 ±28.16 pmol/oocyte-hour (A) and 46.73 ±0.58 pmol/oocyte-hour (B) for PiT1, 2.78 ±0.74 pmol/oocyte-hour for PiT1 $E_{70}K$ (A), 30.99 ±8.67 pmol/oocyte-hour for H_2O injected (A), 44.96 ±0.46 pmol/oocyte-hour for PiT2 (B), 2.36 ±0.56 pmol/oocyte-hour for PiT2 $H_{502}A$ (B), and 3.24 ±0.17 pmol/oocyte-hour for H_2O injected (B).

C The average numbers (±SEM) of blue (infected) cells per dish from three dishes receiving independent precipitates are as follows: 10A1 MLV: 884 ±146 (PiT1), 767 ±42 (PiT1 $E_{70}K$), and 0 (Mock). The *P*-value relative to PiT1 is as follows: PiT1 $E_{70}K$ *P*=0.48.

D-E The average numbers (±SEM) of blue (infected) cells per dish from three dishes receiving independent precipitates are as follows: for 10A1 MLV: 63,940 ±8076 (PiT2), 50,408 ±4005 (PiT2 $H_{502}A$), and 0 (Mock) and for A-MLV: 13,624 ±862 (PiT2), 12,235 ±1189 (PiT2 $H_{502}A$) and 0 (Mock). The *P*-values relative to PiT2 are as follows: PiT2 $H_{502}A$ *P*=0.23 (D) and *P*=0.48 (E).

Figure 4

Investigation of the loop sequence length in PiT family members.

B Data are the mean value of (n) numbers of loops counted ±SEM. The average loop lengths are as follows, L1: 17.1 ±1.5 amino acids (n=9), L2: 19.8 ±0.1 amino acids (n=9), L3: 38.6 ±1.7 amino acids (n=9), L4: 13.3 ±1.2 amino acids (n=7), L5: 9.6 ±1.7 amino acids (n=7), L6: 131.7 ±32.8

amino acids (n=9), L7: 42.9 ±14.7 amino acids (n=9), L8: 24.6 ±2.5 amino acids (n=9), L9: 26.4 ±5.1 amino acids (n=9).

Figure 6

Na^{32}P$_i$ uptake mediated by human PiT2 and truncation mutants analyzed in *X. laevis* oocytes.

Data are the mean value of (n) numbers of oocytes ±SEM. The *P* values for comparison to PiT2 are as follows: PiT2ΔL_{183}-V_{483} P=0.003 (A) and P=0.004 (B), PiT2ΔR_{254}-V_{483} P=0.119 (A) and P=0.553 (B), H_2O P=0.002 (A) and P=0.002 (B). For comparison to H_2O: PiT2ΔL_{183}-V_{483} P=0.011 (A) and P=0.008 (B), PiT2ΔR_{254}-V_{483} P<0.001 (A) and P<0.001 (B). The average $^{32}P_i$ uptakes were 79.61 ±17.74 pmol/oocyte-hour (A) and 44.96 ±0.46 pmol/oocyte-hour (B) for PiT2, 3.93 ±0.44 pmol/oocyte-hour (A) and 8.33 ±2.85 pmol/oocyte-hour (B) for PiT2ΔL_{183}-V_{483}, 47.38 ±6.59 pmol/oocyte-hour (A) and 38.74 ±3.73 pmol/oocyte-hour (B) for PiT2ΔR_{254}-V_{483}, and 2.56 ±0.24 pmol/oocyte-hour (A) and 3.24 ±0.17 pmol/oocyte-hour (B) for H_2O injected oocytes, respectively.
